# Magnesium–Isotope Fractionation in Chlorophyll-a Extracted from Two Plants with Different Pathways of Carbon Fixation (C3, C4)

**DOI:** 10.3390/molecules25071644

**Published:** 2020-04-03

**Authors:** Katarzyna Wrobel, Jakub Karasiński, Andrii Tupys, Missael Antonio Arroyo Negrete, Ludwik Halicz, Kazimierz Wrobel, Ewa Bulska

**Affiliations:** 1 Faculty of Chemistry, Biological and Chemical Research Centre, University of Warsaw, Zwirki i Wigury 101, 02-093 Warszawa, Poland; jkarasinski@chem.uw.edu.pl (J.K.); atupys@cnbc.uw.edu.pl (A.T.); ludwik@gsi.gov.il (L.H.); 2 Chemistry Department, University of Guanajuato, L. de Retana 5, 36000 Guanajuato, Mexico; ma.arroyonegrete@ugto.mx (M.A.A.N.);; 3 Geological Survey of Israel, 32 Y. Leybowitz st., 9692100 Jerusalem, Israel

**Keywords:** Mg-isotope fractionation, chlorophyll, C3 plants, C4 plants, ion chromatography, multiple collector-inductively coupled mass spectrometry

## Abstract

Relatively few studies have been focused so far on magnesium–isotope fractionation during plant growth, element uptake from soil, root-to-leaves transport and during chlorophylls biosynthesis. In this work, maize and garden cress were hydroponically grown in identical conditions in order to examine if the carbon fixation pathway (C4, C3, respectively) might have impact on Mg-isotope fractionation in chlorophyll-a. The pigment was purified from plants extracts by preparative reversed phase chromatography, and its identity was confirmed by high-resolution mass spectrometry. The green parts of plants and chlorophyll-a fractions were acid-digested and submitted to ion chromatography coupled through desolvation system to multiple collector inductively coupled plasma-mass spectrometry. Clear preference for heavy Mg-isotopes was found in maize green parts (∆^26^Mg_plant-nutrient_ 0.65, 0.74 for two biological replicates, respectively) and in chlorophyll-a (∆^26^Mg_chlorophyll-plant_ 1.51, 2.19). In garden cress, heavy isotopes were depleted in green parts (∆^26^Mg_plant-nutrient_ (−0.87)–(−0.92)) and the preference for heavy isotopes in chlorophyll-a was less marked relative to maize (∆^26^Mg_chlorophyll-plant_ 0.55–0.52). The observed effect might be ascribed to overall higher production of energy in form of adenosine triphosphate (ATP), required for carbon fixation in C4 compared to C3, which could reduce kinetic barrier and make equilibrium fractionation prevailing during magnesium incorporation to protoporphyrin ring.

## 1. Introduction

The phenomenon of mass-dependent stable isotope fractionation is well documented for variety of elements in both, abiotic and biotic systems [1]. Analytical data are helpful in assessing biogeochemical cycles of these elements and necessary for better understanding of their isotope-dependent pathways in living organisms [2,3]. Important applications in studies of food provenance/authentication, in archaeometry, forensic science, among others, should also be mentioned [4,5]. Whereas for traditional elements (C, H, O, N, S), stable isotope fractionation measurements can be performed routinely, studies focusing on stable isotopes of non-traditional metals/metalloids are much more challenging due to the less pronounced fractionation and higher mass discrimination [3]. Multiple collector inductively coupled plasma-mass spectrometry (MC ICP-MS) is now accepted as a gold standard in providing reliable isotope fractionation data for metals of geochemical and biological relevance [6,7]. This technique offers efficient ionization, high mass resolution and high-precision measurements with relatively low sample requirement and high throughput. On the other hand, MC ICP-MS is a very complex instrument, sensible to sample matrix composition, memory effects, suffering from instrumental mass bias and spectral interferences [7]. To attain high accuracy and precision, not only instrument operating parameters need to be carefully selected, but primarily the extensive sample clean-up procedure is mandatory. For metals such as Mg, Ca, and Sr, direct coupling of ion chromatography (IC) to MC ICP-MS has been proposed as a feasible alternative simplifying sample preparation [8,9,10]. In the instrumental set-up, column effluent was passed through a self-regenerating suppressor for the replacement of mobile phase anion(s) by OH^–^ and, then, only the column fraction containing metal of interest entered the plasma. This was achieved within the six-port injection valve, where column effluent before and after analyte elution was discarded while bracketing solution was directed to MC ICP-MS [8].

Magnesium is one of the major elements of the Earth’s crust, whose three stable isotopes, ^24^Mg (78.99%), ^25^Mg (10.00%), ^26^Mg (11.01%), present low variation amplitude (δ^26^Mg roughly up to 6‰) [1,5,11]. Even though relatively large mass differences exist among the three isotopes and Mg has no radiogenic isotopes nor redox activity, isotope fractionation measurements are still demanding due to the troublesome spectral interferences and the need for high precision. In line with these requirements, MC ICP-MS provides precision down to 0.10‰ or even better [1,9,12].

Magnesium is an important mineral nutrient in all life domains and considerable attention has been paid to deciphering its isotope fractionation in plants [1,13,14]. In the cited works, isotope fractionation was studied during plant growth, element uptake from soil or nutrient solution and during xylem transport from roots to leaves. According to the actual state-of-the-art, enrichment with heavy isotopes occurs during Mg uptake after which lighter isotopes are preferentially transported to the leaves; however, the observed outcome always depends on the environmental conditions, nutrients availability, externally imposed stress, and other variables [1,13,14]. Noteworthy is that, prior to the clean-up and MC ICP-MS analysis, acid-digestion of the biomass of a specific morphological part or the whole plant is needed and hence contributions from individual biological processes cannot be assessed.

On the other hand, there is a need for better understanding of the processes occurring at intracellular level; in this regard, Mg isotope fractionation during biosynthesis of photosynthetic pigments has been studied [14,15,16,17,18] and approached by theoretical calculations [19]. In the experimental studies performed on chlorophyll extracts both, depletion [1,14,15] and enrichment [16,18] with heavy isotopes have been reported in pigments, relative to leaves or culture medium. It has been proposed that isotope fractionation occurs during insertion of Mg into protoporphyrin IX by magnesium chelatase, with preferential chelation of lighter Mg isotopes within the enzyme backbone preceding metal ion insertion to the coordination site [15].

The experimental data of Mg isotope fractionation in chlorophylls have been obtained for higher plants presenting C3 carbon fixation pathway [14,16], in cyanobacteria [15,17] and marine phytoplankton [18] whereas C4 plants have not yet been considered. Noteworthy is that C4 plants have evolved in dry/hot environments to reduce photorespiration and the risk of dehydration, and to achieve more efficient delivery of CO_2_ in the photosynthesis [20]. The additional step of carbon fixation in C4 with respect to C3 plants relies on the enzymatic conversion of CO_2_ to oxaloacetic acid in mesophyll cells; this four-carbon compound is then transformed to malate and delivered to the bundle sheath cells where CO_2_ is released and re-fixed by enzyme RuBiSco in Calvin cycle [20]. Under conditions of high light intensity, elevated temperatures and limitation of water and/or CO_2_, photosynthesis is more efficient in C4 plants; however, the energy requirement in form of adenosine triphosphate (ATP) is also higher [21]. 

It seems feasible that different energy status in chloroplasts of C3 and C4 plants might have impact on Mg isotope fractionation during chlorophyll synthesis. To verify this assumption, garden cress (C3) and maize (C4) [22] were grown in exactly the same hydroponic conditions and Mg isotope fractionation in chlorophyll-a was assessed by ion chromatography (IC)-MC ICP-MS. Chlorophyll-a fraction was obtained from plant extracts by preparative-scale reversed phase chromatography and the compound identity was confirmed by high resolution mass spectrometry. Possible biological rationale of the obtained results has been proposed.

## 2. Results and Discussion

The main idea of this study was to examine Mg-isotope fractionation in chlorophyll-a extracted from two plants with different carbon fixation systems. Maize and garden cress were selected as C4 and C3 plants, respectively. Multiple factors related to the plant environment, growth stage, morphological part as well as the conditions applied during sample pretreatment, certainly may affect isotope fractionation. To avoid the contribution of these variables, the two plants were cultivated and processed simultaneously in exactly the same growth and experimental conditions. Two biological replicates were prepared for each plant, growing them in June and in September 2019. In such approach, possible changes in Mg-isotope fractionation can be ascribed specifically to the individual plant physiology. In each biological replicate, single pooled extract of chlorophyll-a was obtained for garden cress and for maize, respectively; these extracts were acid digested and appropriately diluted. Five technical replicates were performed taking aliquots from such prepared samples for IC MC ICP-MS measurements (in total 20 analytical runs). In parallel, Mg-isotope fractionation was measured in green parts of the plants harvested from two biological replicates, also applying five technical replicates (20 analytical runs by IC-MC ICP-MS) after the same digestion procedure.

### 2.1. Separation and Characterization of Chlorophyll-a 

Among different protocols proposed for plant pigments extraction, the one previously used for the determination of stable carbon and nitrogen isotope compositions was adopted in this work [23]. As described in Section 3.3, pigments separation was performed by preparative-scale chromatography. Elution of three fractions was observed with diode array spectrophotometric detection, which is evidenced in Figure S1 (Supplementary Materials). In this same Figure S1, absorption spectra acquired in the apex of the first two fractions are presented, suggesting that the fractions I and II corresponded to chlorophyll-b and chlorophyll-a, respectively. Indeed, the absorption maximum for fraction I corresponded to 654 nm and for fraction II, two absorption maxima were observed (less intense at 616 nm and more intense at 665 nm), in agreement with data reported elsewhere [24]. Three fractions were collected from a single run for maize and for garden cress and photographs of these fractions are shown in Figure S1. Electrospray ionization-high resolution mass spectra acquired for fractions I and II (detailed description in Section 3.3.) confirmed the identity of chlorophyll-a and chlorophyll-b, based on the exact mass measurement (mass error 1.7 ppm and 3.9 ppm, respectively) and good agreement between experimental and in silico isotopic patterns (data shown in Figure S1, Supplementary Materials). Most importantly, the above results demonstrate that Mg is preserved in the pigment structure after extraction procedure and after chromatographic separation. It is also noteworthy that chlorophyll-a was more abundant in both plants compared to chlorophyll-b (see chromatogram in Figure S1), in consistency with other reports [15,25]. As to the fraction III, pheophytin-associated ions together with a series of unidentified signals were observed; this fraction was not considered in further experiments.

Total Mg concentration found in the freeze-dried maize biomass corresponded to 2.64 ± 0.04 mg/g in the first harvest (June) and 2.45 ± 0.02 mg/g in the second harvest (September). For garden cress, the respective values were 6.67 ± 0.03 mg/g and 7.45 ± 0.04 mg/g. The primary goal during sample preparation was to obtain the amount of individual chlorophylls (containing bound Mg), enough to perform Mg-isotope ratio measurements by IC-MC ICP-MS. Magnesium in raw pigments extracts accounted for about 2.7–3.2% of total element content in maize and 3.3–3.9% in garden cress. For both plants and two biological replicates, the percentage values were lower than approximately 6% reported previously for wheat (*Triticum aestivum* L.) [14], which should be ascribed principally to non-quantitative extraction/separation protocol and, to less extend, to physiological differences among biological species and shorter growth time used in this work. Further, magnesium was determined in acid-digested pooled chlorophyll fractions; total mass in chlorophyll-a obtained from June experiment was 94 µg for maize and 115 µg for garden cress whereas for September harvest, the respective masses were 88 µg and 95 µg. Based on these data, the solutions for IC-MC ICP-MS analyses were prepared adjusting final magnesium concentration to about 1 mg/L. For chlorophyll-b, Mg content in its pooled fractions for both plants and two biological replicates were always below 20 µg hence insufficient for reliable Mg-isotope measurements. For other metals, potentially interfering with Mg-isotope measurements, total amounts found in four chlorophyll-a fractions (two plants, two harvests) were as follows: 0.31–0.50 µg Zn, 2.67–13.2 µg Ca, 0.43–1.57 µg Na; Al and Fe were detected below quadrupole ICP-MS quantification limit. Low concentrations of all these elements and application of ion chromatography separation ensured interference-free Mg-isotope measurements.

### 2.2. Mg-isotope Fractionation in Chlorophyll-a

The main concern in IC-MC ICP-MS system was to ensure efficient retention of magnesium on the cation exchange column while analyzing acid-digested chlorophyll-a. High acidity of these samples caused partial elution of magnesium in a dead volume (even after dilution suitable for Mg measurements). Systematic experiments performed using Mg standard solution with different additions of nitric acid showed that, for proper Mg retention, nitric acid concentration cannot exceed 0.02 M. To decrease acidity of the samples and to avoid excessive dilution, it was decided to evaporate acid-digests and to adjust acid concentration during re-constitution. As described in Section 3.4, prior to IC-MC ICP-MS measurements, all samples contained about 1 mg/L Mg and 0.02 M nitric acid. The recovery of Mg in the analytical procedure was tested for DSM-3 and Cambridge-1 standards, as well as for chlorophyll-a samples. The obtained results are presented in Table 1, percentage values in the range 78.9%–104% were considered acceptable. It should also be mentioned that differences between δ^26^Mg and δ^25^Mg values measured for DSM-3 and Cambridge-1 standards directly and after analytical procedure were negligible.

**Figure 1:**
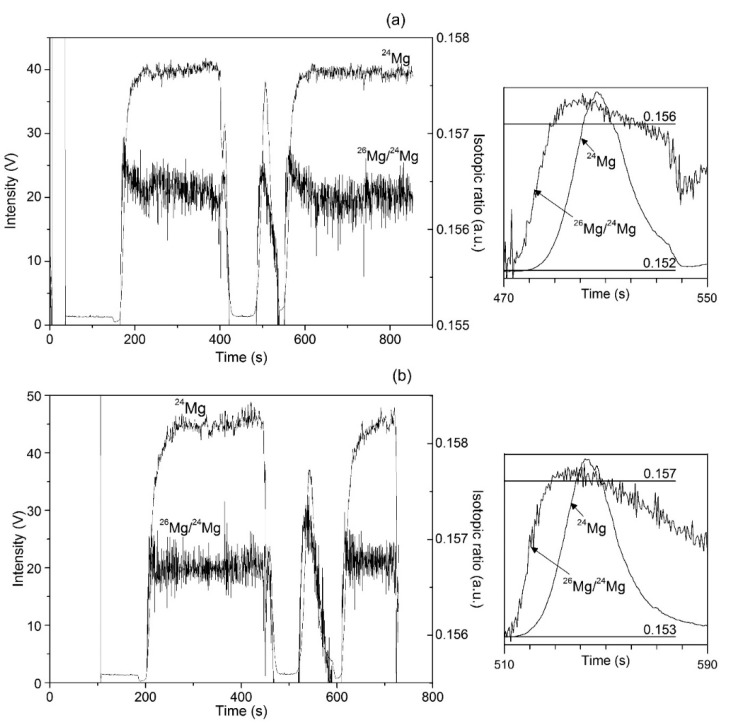
Mg-isotope analysis in chlorophyll-a from garden cress (**a**) and from maize (**b**). Left panel shows multiple collector inductively coupled plasma-mass spectrometry (MC ICP-MS) measurements during chromatographic run (the flat-top peaks are from DSM-3 standard and the signal in the middle corresponds to Mg elution from cation exchange column). Isotopic ratio ^26^Mg/^24^Mg during chromatographic peak elution is presented for the two plants in the right panel.

Typical time-dependent profiles of Mg-isotope measurements by IC-MC ICP-MS in chlorophyll-a extracts are presented in Figure 1 for garden cress (Figure 1a) and for maize (Figure 1b). In the instrumental set-up, mass-bias correction was achieved by referring isotope ratios integrated during Mg elution from the column (transient peak in the middle, left panel in Figure 1) to the average values of the bracket DSM-3 standard (integrated flat regions of the side signals, left panel in Figure 1). The right panel of Figure 1 shows ^26^Mg/^24^Mg isotopic ratio acquired during chromatographic peak elution; important difference between chlorophylls-a from two plants is clearly observed.

**Figure 2:**
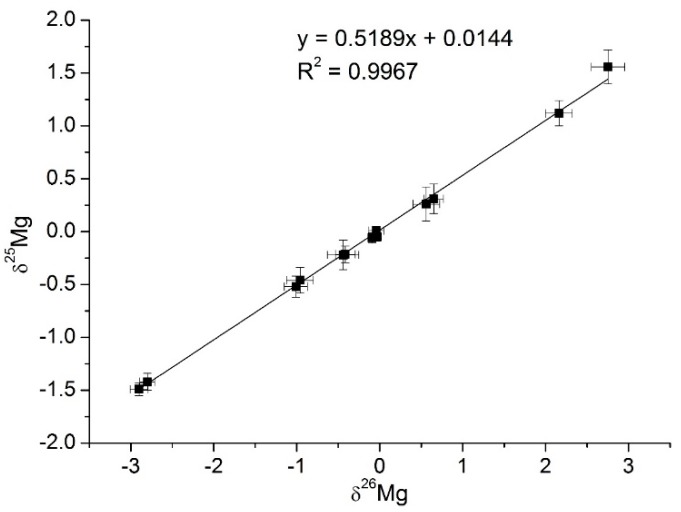
Magnesium–isotope ratios obtained for the samples and standards analyzed in this work; all values are expressed as permille relative to DSM-3 and were calculated using Equation (1) (Section 3.5.). Mean results are presented based on five replicates; the error bars correspond to 2SD. Linear regression fit represents the equilibrium mass-dependent fractionation line.

Once the standards and all samples were analyzed, a three-isotope plot was obtained and is presented in Figure 2; means with respective 2SD values are based on five replicates of each sample. Trend line fitted by linear least square method yielded slope value 0.5190 and *R*^2^ = 0.9967. Good agreement of the slope value with those reported for terrestrial materials (0.5163–0.5181 [12,26]) supports that measured isotope fractionation was consistent with mass-dependent fractionation law. The respective values of δ^26^Mg and δ^25^Mg (relative to DSM-3) are summarized in Table 2 together with several representative data reported previously [9,12,14,16,17,18,27]. At first, sound agreement between results obtained for two biological replicates (June and September experiments) is indicative of good repeatability of the procedure and supports that the obtained data are representative for the biological systems under study. It is also observed in Table 2 that isotope fractionation measured for DSM-3 and Cambridge-1 standards closely match those previously reported [9,12,14], which further proves validity of analytical data obtained in this work. Total Mg concentration found in nutrient solution matched that of added MgCl_2_ (difference < 1%) therefore δ^26^Mg and δ ^25^Mg measured directly in salt were considered valid for nutrient solution; the obtained values were −0.09 ± 0.06‰ and −0.05 ± 0.05‰, respectively. For green parts biomass and for chlorophyll-a, δ^26^Mg and δ^25^Mg values were positive in maize whereas for garden cress these values were negative, which clearly indicates the preference of C4 plant for heavy Mg isotopes. Noteworthy is that negative δ^26^Mg and δ^25^Mg values were reported in leaves of different terrestrial C3 plants, in cyanobacteria and algae (Table 2) in consistency with results obtained in this work for garden cress; however, to the best of our knowledge no data are available for C4 plants.

Pursuing the main objective of this work, changes of isotope composition between nutrient solution, green plant parts, and chlorophyll-a were calculated for each plant and for each biological replicate; the obtained ∆^26^Mg and ∆^25^Mg values are presented in Table 3. While comparing data for nutrient solution and biomass of green plant parts, depletion of heavy isotopes during the 11-day hydroponic growth of garden cress occurred (∆^26^Mg_plant-nutrient_ values −0.87‰ and −0.92‰, for June and September experiments). In contrast, enrichment of maize with heavy isotopes was evident with respective ∆^26^Mg_plant-nutrient_ values 0.74‰ and 0.65‰. According with earlier studies, hydroponically grown plants preferentially incorporate heavy Mg isotopes from nutrient solution yet green parts are relatively lighter than roots because xylem transport of light isotope is favored [1,13,14]. Even though roots were not analyzed in this work, the results obtained suggest differences between two plants in root-to-shoot transport of Mg isotopes in 11-day seedlings. Most importantly, differences in isotopes composition between green parts and chlorophyll-a point to relatively easier incorporation of heavy isotopes during pigment biosynthesis; however, this effect is much more pronounced for maize compared to garden cress (Table 3).

As already mentioned in the introduction and evidenced in Table 2, depending on the biological species as well as the growth time and applied conditions, depletion [1,14,15] or enrichment [16,18] of chlorophyll with heavy Mg isotopes have been reported. Since in this work, the two plants were grown simultaneously in identical conditions, the heavy-isotopes enrichment of maize seedling and its chlorophyll-a in comparison to garden cress, was ascribed to different physiology of these two plants (C4 and C3, respectively). During biosynthesis of chlorophyll, Mg incorporation into protoporphyrin IX is catalyzed by Mg-chelatase via Mg binding to the functional groups present in protein structure; it has been proposed that binding of lighter Mg isotopes might be favored at this stage [15]. On the other hand, ATP hydrolysis providing energy for labilization of Mg-coordinated water is required during Mg internalization into the coordination center of protoporphyrin. The requirement for ATP during CO_2_ fixation is greater in C4 with respect to C3 [20] and it seems possible that higher energy status in C4 could reduce kinetic barrier during Mg incorporation to protoporphyrin IX and the observed heavier signature of chlorophyll-a in maize might be ascribed to equilibrium- rather than kinetic (enzymatic) isotope fractionation. It should also be emphasized that the role of Mg in higher plants is not limited to its presence in chlorophyll. Magnesium homeostasis is complex and involves multiple enzymatic and chemical processes [28] potentially affecting isotope fractionation; therefore, the results obtained in this work cannot be interpreted exclusively within the context of chlorophylls. It seems particularly relevant that ATP itself binds magnesium with high affinity and this binding is a requisite for biological activity of ATP; furthermore, ATP-synthase depends on Mg and incorporation of paramagnetic ^25^Mg has been related with higher enzymatic activity [29]. Overall, different Mg-isotope fractionation observed in this study for two plants with different energy requirements and ATP production rates suggest that Mg involvement in ATP-related processes might be responsible for isotope fractionation in chlorophyll and in other plants compartments.

## 3. Materials and Methods

### 3.1. Reagents and Standards

All chemicals were of analytical reagent grade. Deionized water (18.2 MΩ cm, Water PRO^TM^ PS, Labconco, Kansas, MO, USA), HPLC-grade methanol, isopropanol, dioxane and acetone from Sigma (Milwaukee, WI, USA), were used throughout.

Ultrapure nitric acid and hydride peroxide were from J.T. Baker (Instra-Analyzed Plus). Multi-elemental standard solution for ICP-MS containing Li, Al, V, Cr, Mn, Co, Ni, Cu, Rb, Sr, Mo, Ag, Cd, Ba, Tl, Pb, Bi, U at 9.8 mg L^−1^, and Be, Fe, As, Se, Zn at 100 mg L^−1^, was Merck ICP VI (Merck, Darmstadt, Germany).

Isotope fractionation was measured relative to Mg standard solution DSM-3 (Dead Sea Magnesium Ltd., Israel) and Cambridge-1 reference material with δ^26^Mg value relative to DSM-3 −2.58 ± 0.14 (2σ) [30] was used.

Sodium hypochlorite and methanesulfonic acid were Sigma reagents; the Hoagland modified nutrient solution containing Ca(NO_3_)_2_ (0.35mM), CaCl_2_ (2.1 mM), MgCl_2_ (0.91 mM), KH_2_PO_4_ (0.97 mM), KNO_3_ (1.22 mM), H_3_BO_3_ (23 µM), MnCl_2_ (3.9 µM), MoO_3_ (23 μM), Fe(NO_3_)_3_ (10 μM), Zn(NO_3_)_2_ (0.37 μM), CuSO_4_ (0.44 μM), pH 5.8, was prepared also from Sigma reagents [31].

### 3.2. Plants Growth and Chlorophyll Extraction

Seeds of garden cress (*Lepidium sativum* cv. *Ogrodowa*) and of maize (*Zea mays*) were purchased in the garden-specialized markets in Poland and in Mexico, respectively. Once surface sterilized with 3% *m*/*v* sodium hypochlorite for 20 min, seeds were soaked in deionized water for 60 min and then, were hydroponically germinated and grown, using modified Hoagland’s nutrient. The trays with plant cultures were placed in the growth chamber without light for the first 48 h and then, the twelve-hours cycles of light (6am–6pm, 25 °C, 60.8 µmol m^-2^ s^-1^ photon irradiance from fluorescent lamp) and darkness (6pm–6am, 18 °C) were applied; relative humidity was maintained at around 60%. The trays were moved within the chamber each day to ensure these same conditions for all cultures. Green parts were harvested after 11 days. Figure S2 in the Supplementary Materials shows photo of the cultures in the growth chamber just before harvesting. For each plant type, the obtained biomass was rinsed twice with deionized water, homogenized immediately by grinding in liquid nitrogen and freeze-dried. The growth of two plants was performed twice: in June and in September 2019, respectively (two biological replicates).

Pigments extraction was carried out as described elsewhere [23] yet with slight modifications. At each stage, the samples were protected from light to avoid chlorophyll degradation. In brief, 100 mL of methanol were added to 4g of the freeze-dried biomass and after vortex, the mixture was kept at 4 °C for 24 h. A solid residue was eliminated by filtration (Whatman 541); the filter was rinsed twice with 5 mL portions of methanol. Chlorophylls were precipitated from the filtrate by addition of dioxane and deionized water, yielding the composition of methanol:dioxane:water 10:1:1 (*v*/*v*). To complete precipitation, the samples were left at −20 °C for 24 h and filtered. The chlorophyll fraction was rinsed with two 5-mL portions of the solvent mix and finally dissolved in 10 mL of acetone. General scheme of the procedure with photographs taken at different stages of the procedure are presented in Figure S3 (Supplementary Material).

Two extracts corresponding to two harvests (June and September 2019) were obtained for each plant.

### 3.3. Purification of Chlorophyll-a and High-resolution Mass Spectrometry Analysis

Separation of chlorophylls in acetone extracts was performed using an Agilent 1200 high performance liquid chromatograph with diode array detector and preparative column Zorbax 300 SB-C18 (250 × 9.4 mm, 5 µm) (Agilent Technologies, Santa Clara, CA, USA). Manual injection was performed via externally mounted six-port injection valve (Rheodyne) with the injection loop of 500 µL. Column was maintained at 40 °C, methanol:isopropanol (1:1, *v*/*v*) was used as a mobile phase at 2.5 mL/min flow rate. Chromatograms were registered with detection at 700 nm and 663 nm and chlorophyll-a fraction was collected between 4.4 min and 5.4 min of the chromatographic run. The fractions collected for one harvest, were pooled and evaporated (SpeedVac, (Vacufuge plus, Eppendorf AG, Hamburg, Germany) 45 °C); this was achieved by partial evaporation of the respective column fractions in a series of Eppendorf tubes and successive combination of aliquots to finally obtain solid chlorophyll-a from each plant in a single tube. Two tubes per plant were obtained, each of them corresponding to different harvest (June and September 2019).

High-resolution mass spectra of the chlorophyll fractions were acquired using maXis impact electrospray - quadrupole-time of flight mass spectrometer equipped with Data Analysis 4.1 (Bruker Daltonics, Bremen, Germany). For this purpose, chromatographic fractions were collected from a single run and appropriately diluted with 1% formic acid. During direct infusion, the lock-mass standard (sodium formate, *m/z* 1221.9906) was applied in the ion source, ESI was operated in positive mode with ion spray voltage 4500 V, end plate offset 500 V, dry gas 4 L/min, drying temperature 180 °C and nebulizing gas pressure 0.4 bar; the *m*/*z* range was from 50 to 1250.

### 3.4. Samples Digestion and ICP-MS Determination of Elements

For total Mg determination in the freeze-dried biomass and in acetone extract, respectively, 50 mg and 0.5 mL aliquots were digested in Milestone Ethos One Microwave Digestion System, using 5 mL of concentrated nitric acid and the following program: 15 min ramping to 180 °C and 40 bar, than hold for 10 min. The samples were appropriately diluted and introduced to quadrupole ICP-MS (Perkin Elmer NexION 300D, Waltham, MA, USA); the ^26^Mg was monitored. Magnesium was also determined in nutrient solution, after appropriate dilution.

For total elements determination and isotope measurements in chlorophyll-a fraction, 1 mL of concentrated nitric acid was added to the Eppendorf tube containing respective evaporated and pooled column fraction, and the sample was left overnight at room temperature. Next, the tube content was transferred to a PTFE vessel, 3 mL of concentrated nitric acid were added, and microwave-assisted digestion was performed in similar conditions as above: 15 min ramping to 180 °C and 40 bar, then held for 25 min. Once cooled to room temperature, 1 mL of hydrogen peroxide was added, and heating program was repeated. The obtained solution was quantitatively transferred to Falcon tube which was placed in boiling water until complete evaporation (about 7 h). The solid residue was dissolved in appropriate volume of water with small addition of nitric acid to obtain final magnesium concentration of about 1 mg/L and acid concentration of 0.02 M. In parallel, 50 mg aliquots of the freeze-dried biomass from each harvest, DSM-3 (1000 µg/L of Mg), Cambridge-1 (1000 µg/L of Mg), and blank were processed. To examine possible Mg loss during above operations, 50 µL aliquot was taken after each digestion step and total Mg was determined by quadrupole ICP-MS. Additionally, elements potentially affecting Mg isotope measurements were determined in the final solution (^23^Na, ^27^Al, ^44^Ca, ^57^Fe, ^68^Zn).

### 3.5. Ion Chromatography-dry Plasma MC ICP-MS Analysis

Previously described instrumental setup was adopted [9]; the general scheme is presented in Figure S4 (Supplementary Materials). In brief, 25 µL sample aliquot was mixed in autosampler (AS-D) with a mobile phase and introduced to an ion chromatograph (Dionex ICS-5000+, Thermo Scientific, Dreieich, Germany), in which inorganic cation exchange column IonPackC16 (3 × 250 mm, 5 µm) with a guard column IonPac CG16 (3 × 50 mm) were mounted. The column was kept at 40 °C, methanesulfonic acid 51 mM was used as a mobile phase at the flow 0.36 mL/min. The column effluent was passed through the self-regenerating suppressor (CDRS 600 2 mm) operated at 20 °C and then, was carried through a conductivity detector to a six-port valve. By so doing, only the column fraction corresponding to Mg elution (from 7.0 min to 9.5 min) was directed to the plasma while for the rest of chromatographic run, column effluent was discarded and bracketing solution (DMS-3) was pumped toward desolvation nebulizer unit at the flow rate 0.36 mL/min. Aridus II desolvation nebulizer (CETAC Technologies, Omaha, NE, USA) was operated setting spray chamber and desolvator temperatures at 120 °C and 160 °C, respectively; the flow rate of sweep gas (Ar) was about 7.50 L/min and the flow rate of nebulizer gas (Ar) was 0.50 L/min. A model “Plasma II” multi collector high resolution mass spectrometer equipped with 16 Faraday cups was used (Nu Instruments, Wrexham, UK). Plasma was operated at RF power of 1300 W, coolant gas flow 13.6 L/min and auxiliary gas flow 1.17 L/min; nickel interface cones were used. Three collectors (L-4, H-2, H-8) were used for measurements of magnesium isotopes 24, 25, and 26, respectively, with integration time of 0.5 s. For each isotope, magnesium eluting from the column was registered as a transient peak and total area was integrated. In contrast, Mg from bracketing solution produced two flat-top peaks (before and after Mg eluting from the column) and signals for each isotope were integrated from the flat region. 

Mg–isotope fractionation in the analyzed samples was calculated from the following equation, in which ^x^Mg corresponds to the signal of ^25^Mg or ^26^Mg:

(1) δxMg (‰)=((M xgM 24g)sample((M xgM 24g)DSM3(1)+(M xgM 24g)DSM3(2))/2−1)×1000

### 3.6. Statistical Analysis

All quadrupole ICP-MS and IC-MC ICP-MS measurements were carried out in five replicates; descriptive statistics was performed to obtain mean values and respective standard deviations (Microsoft Excel 2016, Microsoft, Munich. Germany).

## 4. Conclusions

The original idea of this work was to pursue possible dissimilarities in Mg-isotope fractionation in chlorophyll-a from two plants presenting different pathways of carbon fixation. For unambiguous association of the observed effects with specific plant physiology, both plants were grown simultaneously, in identical hydroponic conditions and two biological replicates were prepared and analyzed. Chlorophyll identity in the separated plant fractions was confirmed by high-resolution mass spectrometry. Mg-isotope measurements were performed adopting previously established IC MC-ICP-MS system; however, complete elimination of organic matter (HNO_3_-H_2_O_2_ microwave digestion) and adjustment of nitric acid concentration to 0.02 M (evaporation and re-constitution of the digest) were found necessary to achieve the desired retention of Mg in chromatographic process. Noteworthy is that isotope fractionation measured in all samples and standards obeyed the mass-dependent fractionation law, confirming the consistency of analytical results. As to the biological relevance, the obtained results revealed clear preference of C4 plant (maize) for heavy Mg–isotopes in green parts and in chlorophyll-a as compared to C3 plant (garden cress). This finding was ascribed to greater energy requirements and ATP production rates in C4 plant which might reduce energy barrier during Mg incorporation to the protoporphyrin IX. More generally, it can be speculated that Mg involvement in various ATP-related processes could be responsible for different isotope fractionation not only in chlorophyll but also in other compartments of C3 and C4 plants. As far as the authors are aware, this is the first study comparing Mg-isotope fractionation in C4 and C3 plants and, to get further insight, in future experiments two different conditions of temperature, humidity and illumination will be applied during growth: one typical for C3 plants, and another one resembling hot/dry climate - optimal for C4 plants.

## Figures and Tables

**Table 1:** Magnesium recovery in analytical procedure (acid-digestion, evaporation, reconstitution and IC-MC ICP-MS). Initial concentration of Mg was set at 1000 µg/L; for chlorophyll-a samples-adjustment was based on quadrupole ICP-MS determination (means and RSD values based on five replicates).

Sample	Peak Area (mean) µS min	RSD, %	Recovery, %
DSM-3 (directly)	1.62	1.04	-
DSM-3	1.39	1.06	85.4
Cambridge-1	1.68	0.06	104
Maize chlorophyll-a	1.28	0.23	78.9
Garden cress chlorophyll-a	1.50	0.42	92.2

**Table 2:** Magnesium–isotope ratio values obtained for the samples analyzed in this work (mean ± 2SD, based on five replicates) and selected values reported in the literature.

Sample	δ^26^Mg, ‰	δ^25^Mg, ‰
This Work		
Maize-June	0.65 ± 0.12	0.31 ± 0.14
Maize-September	0.56 ± 0.16	0.26 ± 0.10
Garden cress-June	−0.96 ± 0.6	−0.46 ± 0.12
Garden cress-September	−1.01 ± 0.14	−0.52 ± 0.10
MgCl_2_ (nutrient solution)	−0.09 ± 0.06	−0.05 ± 0.05
Maize chlorophyll-a-June	2.16 ± 0.16	1.12 ± 0.12
Maize chlorophyll-a-September	2.75 ± 0.20	1.56 ± 0.16
Garden cress chlorophyll-a-June	−0.414 ± 0.12	−0.21 ± 0.08
Garden cress chlorophyll-a-September	−0.440 ± 0.19	−0.22 ± 0.14
DSM-3-June	−0.04 ± 0.01	0.09 ± 0.02
DSM-3-September	−0.03 ± 0.02	0.05 ±0.04
Cambridge-1-June	−2.80 ±0.09	−1.42 ± 0.08
Cambridge-1-September	−2.89 ± 0.11	−1.49 ± 0.06
**Reported Values**		
DSM-3 [12]	0.01 ± 0.14	0.00 ± 0.09
DSM-3 [9]	−0.04 ± 0.17	
Cambridge-1 [14]	−2.60 ± 0.14	−1.34 ± 0.07
Cambridge [12]	−2.71 ± 0.18	−1.39 ± 0.08
Clover leaf [13]	−0.61 ± 0.14	−0.31 ± 0.07
Wheat leaf [14]	0.11	0.05
Wheat chlorophyll [14]	(−0.34)–(−0.58)	(−0.18)–(−0.27)
Cyanobacteria chlorophyll-a [17]	(−0.12)–(−2.13)	(−0.09)−(−0.62)
English Ivy leaf [16]	(−0.510)–(−0.644)	(−0.277)–(−0.343)
English Ivy chlorophyll [16]	−0.182 ± 0.145	−0.099 ± 0.082
Commercial chlorophyll-a from different plants and algae [18]	1.82–2.76	0.93–1.72
Spinach chlorophyll-a [27]	−1.451 ± 0.098	−0.741 ± 0.062

**Table 3:** Mg-isotope fractionation observed during 11-day plants growth and chlorophyll-a biosynthesis.

Isotope Fractionation	Maize (C4)		Garden Cress (C3)	
	June	September	June	September
∆^26^Mg_plant-nutrient_, ‰	0.74	0.65	−0.87	−0.92
∆^25^Mg_plant-nutrient_, ‰	0.36	0.31	−0.41	−0.47
∆^26^Mg_chlorophyll-plant_, ‰	1.51	2.19	0.55	0.52
∆^25^Mg_chlorophyll-plant_, ‰	0.81	1.30	0.25	0.30

## Supplementary Materials

Figure S1. Separation of chlorophyll-a by preparative RP liquid chromatography, confirmation of its identity by high resolution mass spectrometry and UV/Vis absorption spectra (photographs at different procedural stages included); Figure S2. Photo of plants cultures in the growth chamber taken before harvesting; Figure S3. Outline of chlorophylls extraction and photographs taken at different stages of the procedure; Figure S4. Schematic presentation of instrumental set-up for Mg-isotope measurements.
