# Supplementary material for: Magnesium–Isotope Fractionation in Chlorophyll-a Extracted from Two Plants with Different Pathways of Carbon Fixation (C3, C4)
Source: Molecules. 2020 Apr 3;25(7):1644. doi: 10.3390/molecules25071644 (PMC7181255; doi:10.3390/molecules25071644)
Supplement: Supplementary file 1 [file molecules-25-01644-s001.zip › molecules-743769-supplementary.pptx]

## Slide 1
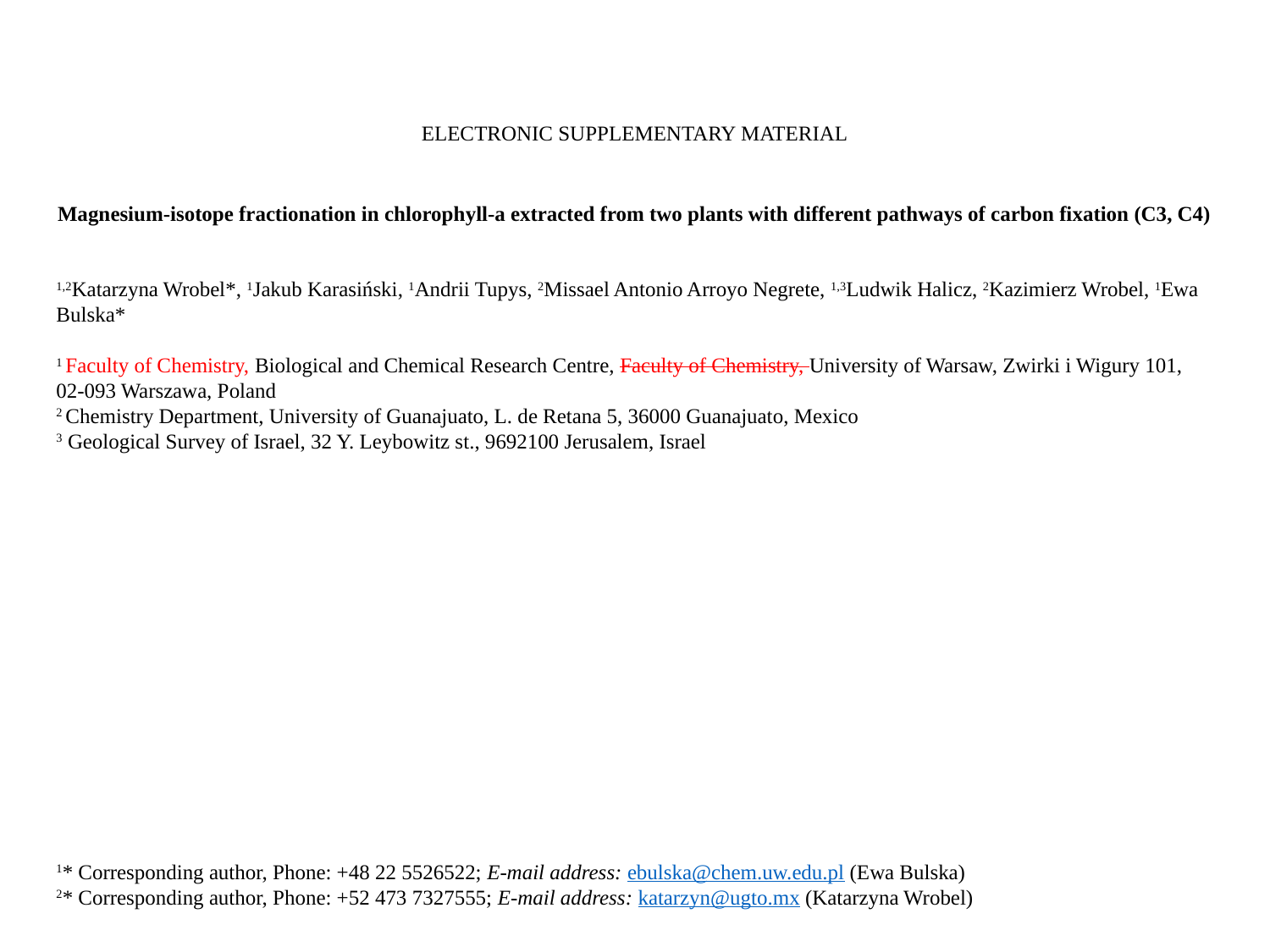

ELECTRONIC SUPPLEMENTARY MATERIAL
Magnesium-isotope fractionation in chlorophyll-a extracted from two plants with different pathways of carbon fixation (C3, C4)
1,2Katarzyna Wrobel*, 1Jakub Karasiński, 1Andrii Tupys, 2Missael Antonio Arroyo Negrete, 1,3Ludwik Halicz, 2Kazimierz Wrobel, 1Ewa Bulska*
1 Faculty of Chemistry, Biological and Chemical Research Centre, Faculty of Chemistry, University of Warsaw, Zwirki i Wigury 101, 02-093 Warszawa, Poland
2 Chemistry Department, University of Guanajuato, L. de Retana 5, 36000 Guanajuato, Mexico
3 Geological Survey of Israel, 32 Y. Leybowitz st., 9692100 Jerusalem, Israel
1* Corresponding author, Phone: +48 22 5526522; E-mail address: ebulska@chem.uw.edu.pl (Ewa Bulska)
2* Corresponding author, Phone: +52 473 7327555; E-mail address: katarzyn@ugto.mx (Katarzyna Wrobel)

## Slide 2
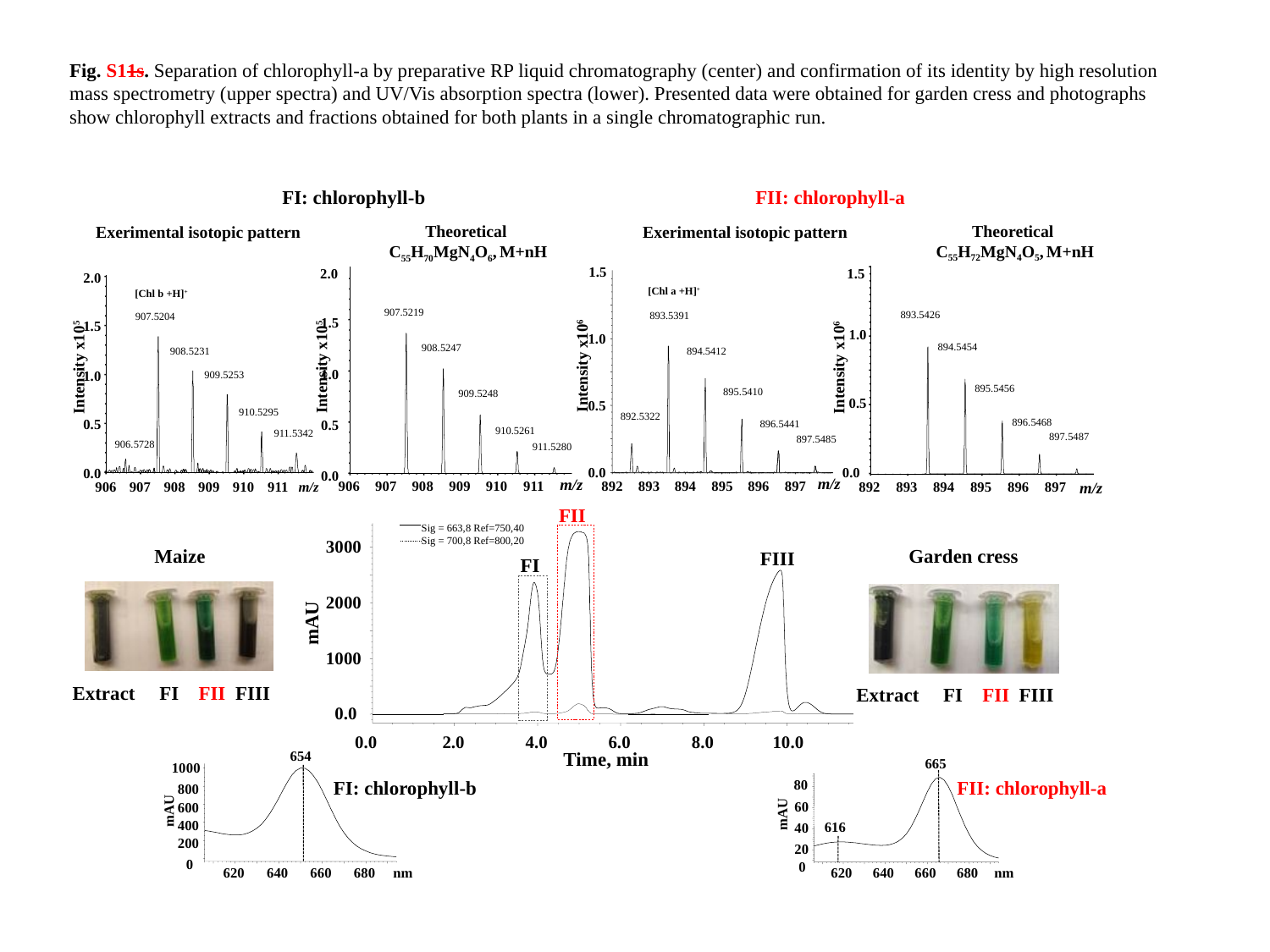

Fig. S11s. Separation of chlorophyll-a by preparative RP liquid chromatography (center) and confirmation of its identity by high resolution mass spectrometry (upper spectra) and UV/Vis absorption spectra (lower). Presented data were obtained for garden cress and photographs show chlorophyll extracts and fractions obtained for both plants in a single chromatographic run.
FI: chlorophyll-b
FII: chlorophyll-a
Exerimental isotopic pattern
Exerimental isotopic pattern
Theoretical
C55H72MgN4O5, M+nH
Theoretical
C55H70MgN4O6, M+nH
1.5
893.5391
1.0
894.5412
895.5410
0.5
892.5322
896.5441
897.5485
0.0
m/z
892
893
894
895
896
897
Intensity x106
1.5
893.5426
1.0
894.5454
895.5456
0.5
896.5468
897.5487
0.0
892
893
894
895
896
897
m/z
Intensity x106
2.0
907.5219
1.5
908.5247
Intensity x105
1.0
909.5248
0.5
910.5261
911.5280
0.0
m/z
906
907
908
909
910
911
2.0
[Chl b +H]+
907.5204
1.5
908.5231
Intensity x105
1.0
909.5253
910.5295
0.5
911.5342
906.5728
0.0
906
907
908
909
910
911
m/z
[Chl a +H]+
FII
Sig = 663,8 Ref=750,40
Sig = 700,8 Ref=800,20
3000
2000
1000
0.0
0.0
2.0
4.0
6.0
8.0
10.0
Time, min
mAU
Maize
Garden cress
FIII
FI
Extract FI FII FIII
Extract FI FII FIII
654
1000
800
600
mAU
400
200
0
620
640
660
680
nm
665
80
60
mAU
40
20
0
620
640
660
680
nm
616
FII: chlorophyll-a
FI: chlorophyll-b

## Slide 3
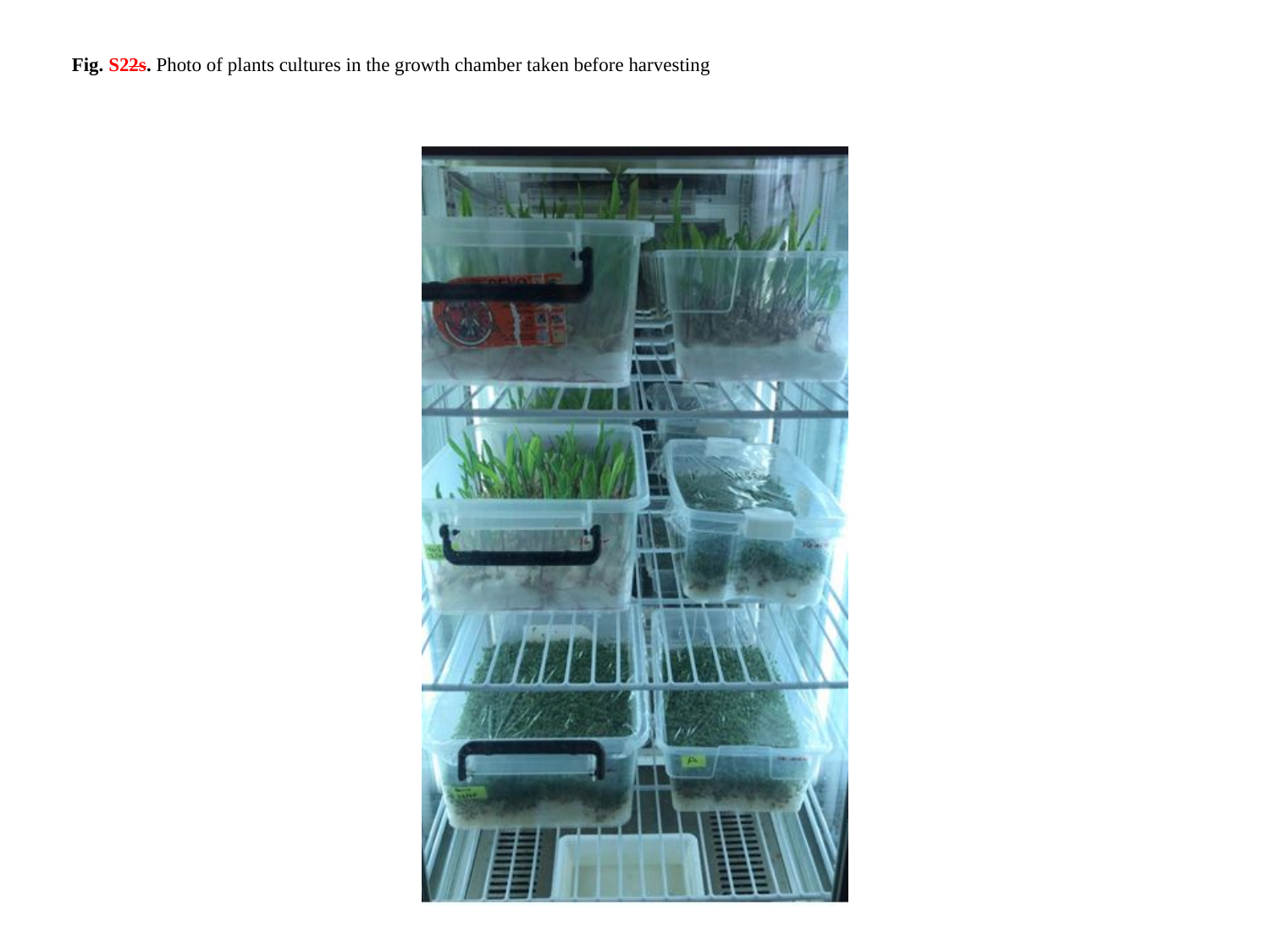

Fig. S22s. Photo of plants cultures in the growth chamber taken before harvesting

## Slide 4
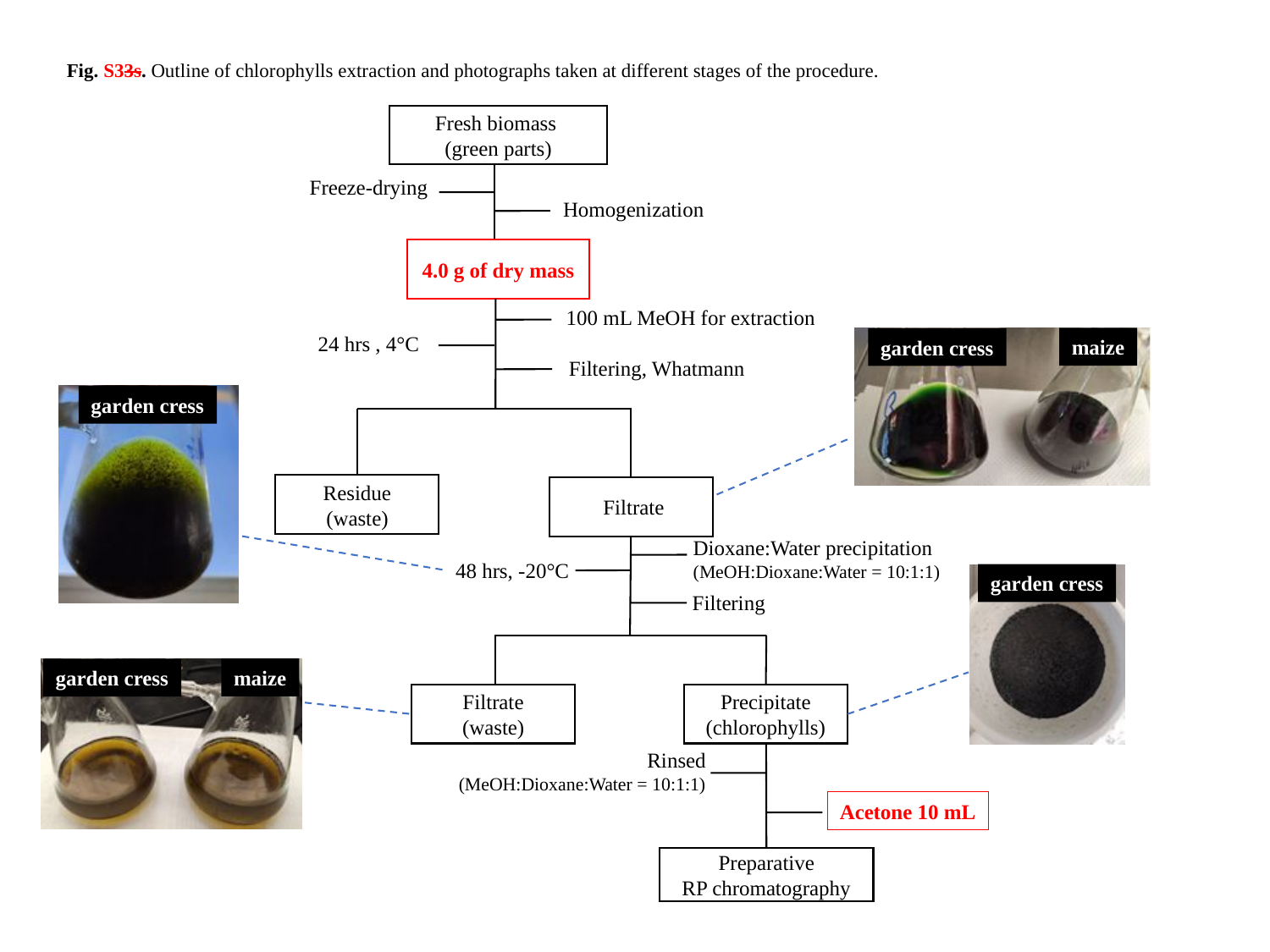

Fig. S33s. Outline of chlorophylls extraction and photographs taken at different stages of the procedure.
Fresh biomass
(green parts)
Freeze-drying
Homogenization
4.0 g of dry mass
100 mL MeOH for extraction
24 hrs , 4°C
Filtering, Whatmann
Residue
(waste)
 Filtrate
Dioxane:Water precipitation
(MeOH:Dioxane:Water = 10:1:1)
48 hrs, -20°C
Filtering
Filtrate
(waste)
Precipitate
(chlorophylls)
 Rinsed
(MeOH:Dioxane:Water = 10:1:1)
Preparative
RP chromatography
Acetone 10 mL
maize
garden cress
garden cress
garden cress
maize
garden cress

## Slide 5
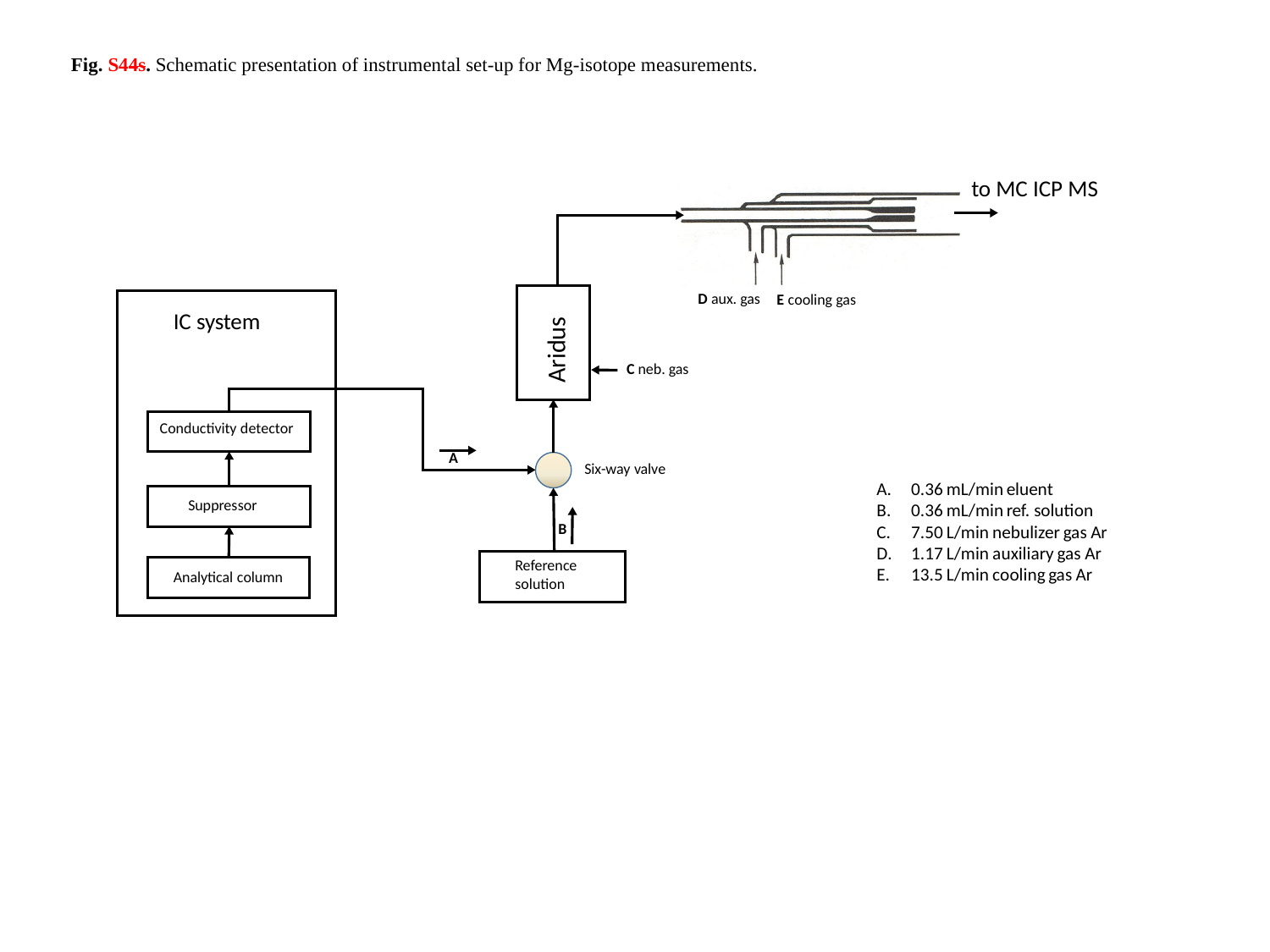

Fig. S44s. Schematic presentation of instrumental set-up for Mg-isotope measurements.
